# Improving clinical paediatric research and learning from COVID-19: recommendations by the Conect4Children expert advice group

**DOI:** 10.1038/s41390-021-01587-3

**Published:** 2021-06-07

**Authors:** Athimalaipet V. Ramanan, Neena Modi, Saskia N. de Wildt, c4c Learning from COVID-19 Group, Beate Aurich, Sophia Bakhtadze, Francisco J. Bautista Sirvent, Fernando Cabañas, Lisa Campbell, Michaela Casanova, Philippa Charlton, Wallace Crandall, Irmgard Eichler, Laura Fregonese, Daniel B. Hawcutt, Pablo Iveli, Thomas Jaki, Bosanka Jocic-Jakubi, Mats Johnson, Florentina Kaguelidou, Bülent Karadag, Lauren E. Kelly, Ming Lim, Carmen Moreno, Eva Neumann, Cécile Ollivier, Mehdi Oualha, Genny Raffaeli, Maria A. Ribeiro, Emmanuel Roilides, Teresa de Rojas, Alba Rubio San Simón, Nicolino Ruperto, Maurizio Scarpa, Matthias Schwab, Angeliki Siapkara, Yogen Singh, Anne Smits, Pasquale Striano, Silvana A. M. Urru, Marina Vivarelli, Saskia de Wildt, Zorica Zivkoviz

**Affiliations:** 1 University Hospitals Bristol NHS Foundation Trust, Bristol, UK; 2 Translational Health Sciences, University of Bristol, Bristol, UK; 3 Section of Neonatal Medicine, Department of Public Health and Primary Care, Imperial College London, London, UK; 4 Chelsea and Westminster NHS Foundation Trust, London, UK; 5 Department of Pharmacology and Toxicology, Radboud Institute for Health Sciences, Radboudumc, Nijmegen, The Netherlands; 6 Intensive Care and Department of Paediatric Surgery, Erasmus MC Sophia Children’s Hospital, Rotterdam, The Netherlands; 7 Robert Debré University Hospital, Paris, France; 8 Tbilisi State Medical University, Tbilisi, Georgia; 9 Hospital Niño Jesús, Madrid, Spain; 10 Quironsalud Madrid University Hospital, Madrid, Spain; 11 Biomedical Research Foundation, La Paz University Hospital-IDIPAZ, Madrid, Spain; 12 Medicines and Healthcare Products Regulatory Agency MHRA, London, UK; 13 Fondazione IRCCS Istituto Nazionale dei Tumori di Milano, Milan, Italy; 14 UCB Biopharma, Anderlecht, Belgium; 15 Eli Lilly and Company, Indianapolis, IN USA; 16 European Medicine Agency EMA, Amsterdam, The Netherlands; 17 Department of Women’s and Children’s Health, University of Liverpool, Liverpool, UK; 18 NIHR Alder Hey Clinical Research Facility, Alder Hey Children’s Hospital, Liverpool, UK; 19 Bayer Aktiengesellschaft, Barmen, Germany; 20 Department of Mathematics and Statistics, Lancaster University, Lancaster, UK; 21 MRC Biostatistics Unit, University of Cambridge, Cambridge, UK; 22 Clinical Center Nis, Pediatric Clinic, Child Neurology Department, Belgrade, Serbia; 23 Sultan Qaboos University Hospital, Department of Pediatric Neurology, Muscat, Oman; 24 Gillberg Neuropsychiatry Centre, Sahlgrenska Academy, Gothenburg University, Gothenburg, Sweden; 25 Clinical Investigations Center, CIC1426, Robert Debré Hospital, Assistance Publique des Hôpitaux de Paris (APHP), Paris, France; 26 Université de Paris, Paris, France; 27 Marmara University Faculty of Medicine, Istanbul, Turkey; 28 Department of Pediatrics and Child Health, University of Manitoba, Children’s Hospital Research Institute of Manitoba, Winnipeg, Canada; 29 Evelina London Children’s Hospital, Guys and St Thomas’ NHS Foundation trust, London, UK; 30 Department Women and Children’s Health, Faculty of Life Sciences and Medicine, King’s College London, London, UK; 31 Department of Child and Adolescent Psychiatry, Institute of Psychiatry and Mental Health, Hospital General Universitario Gregorio Marañón, School of Medicine, Universidad Complutense, IiSGM, CIBERSAM, Madrid, Spain; 32 Dr. Margarete Fischer-Bosch-Institute of Clinical Pharmacology, Stuttgart, Germany; 33 Aparito, Wrexham, United Kingdom; 34 EA7323, University of Paris, Paris, France; 35 Pediatric Intensive Care Unit, AP-HP, Necker Hospital, Paris, France; 36 Department of Clinical Sciences and Community Health, University of Milan, Milan, Italy; 37 NOVA Medical School, Faculdade de Ciências Médicas, Universidade Nova de Lisboa, Lisboa, Portugal; 38 Aristotle University of Thessaloniki, Thessaloniki, Greece; 39 Hippokration Hospital, Athens, Greece; 40 Children’s University Hospital Niño Jesús, Madrid, Spain; 41 IRCCS Istituto Giannina Gaslini, Genova, Italy; 42 Regional Coordinating Center for Rare Diseases, University Hospital, Udine, Italy; 43 Department of Clinical Pharmacology, University Hospital, Tübingen, Germany; 44 Cambridge University Hospitals NHS Foundation Trust, Cambridge, UK; 45 University of Cambridge School of Clinical Medicine, Cambridge, UK; 46 Neonatal intensive care unit, University Hospitals Leuven, Leuven, Belgium; 47 Department of Development and Regeneration, KU Leuven, Leuven, Belgium; 48 University of Genova, Genova, Italy; 49 Chiara Hospital Trento, Trento, Italy; 50 Division of Nephrology and Dialysis, Department of Pediatric Subspecialties, Bambino Gesù Pediatric Hospital IRCCS, Rome, Italy; 51 Children’s Hospital for Lung Diseases & Tb Clinical Center Dr Dragiša Mišovic, Belgrade, Serbia; 52 Faculty of Pharmacy Novi Sad, Business Academy, Novi Sad, Serbia

## Abstract

**Background:**

The COVID-19 pandemic has had a devastating impact on multiple aspects of healthcare, but has also triggered new ways of working, stimulated novel approaches in clinical research and reinforced the value of previous innovations. Conect4children (c4c, www.conect4children.org) is a large collaborative European network to facilitate the development of new medicines for paediatric populations, and is made up of 35 academic and 10 industry partners from 20 European countries, more than 50 third parties, and around 500 affiliated partners.

**Methods:**

We summarise aspects of clinical research in paediatrics stimulated and reinforced by COVID-19 that the Conect4children group recommends regulators, sponsors, and investigators retain for the future, to enhance the efficiency, reduce the cost and burden of medicines and non-interventional studies, and deliver research-equity.

**Findings:**

We summarise aspects of clinical research in paediatrics stimulated and reinforced by COVID-19 that the Conect4children group recommends regulators, sponsors, and investigators retain for the future, to enhance the efficiency, reduce the cost and burden of medicines and non-interventional studies, and deliver research-equityWe provide examples of research innovation, and follow this with recommendations to improve the efficiency of future trials, drawing on industry perspectives, regulatory considerations, infrastructure requirements and parent–patient–public involvement. We end with a comment on progress made towards greater international harmonisation of paediatric research and how lessons learned from COVID-19 studies might assist in further improvements in this important area.

## Introduction

Coronavirus-induced infective disease (COVID-19) has had a devastating impact on multiple aspects of healthcare. However, the pandemic has also triggered new ways of working, stimulated novel approaches in clinical research and reinforced the value of previous innovations.

Conect4children (c4c, www.conect4children.org) is a large collaborative European network that aims to facilitate the development of new medicines for paediatric populations. It is a new collaboration made up of 35 academic and 10 industry partners from 20 European countries, more than 50 third parties, and around 500 affiliated partners. The onset of the pandemic resulted in many c4c partners having to contend with abrupt challenges to their clinical trial operations. These affected access to patients, laboratories and study materials, and availability of clinical research staff. This led many groups to implement novel ways of working to ensure paediatric trials continued to a high standard.

Most authors are member of conect4children expert groups, which include most paediatric subspecialties, trial methodology areas as well as parent patient involvement. These groups provide advice to academia and industry to design innovative paediatric clinical trials. Bringing the expertise of these experts together with experts from the regulatory agencies provided us with the opportunity to present a Europe-wide multidisciplinary COVID-19 experience and suggestions for future innovative trials.

Here, we summarise aspects of clinical research in paediatrics stimulated and reinforced by COVID-19 that we recommend regulators, sponsors and investigators retain for the future, to enhance the efficiency, reduce the cost and burden of medicines and non-interventional studies, and deliver research-equity. We will begin by providing examples of research innovation, and follow this with recommendations to improve the efficiency of future trials, drawing on industry perspectives, regulatory considerations, infrastructure requirements and parent–patient–public involvement. We end with a comment on progress made towards greater international harmonisation of paediatric research and how lessons learned from COVID-19 studies might assist in further improvements in this important area.

## Innovation in paediatric COVID-19 studies: the RECOVERY trial as a paradigm for the future

The long-standing desire of paediatricians to ensure children benefit from participation in clinical trials to the same extent as adults was realised by the UK RECOVERY trial for COVID-19 therapies (https://www.recoverytrial.net).

RECOVERY investigators ensured that children, including infants, were included from an early stage of the trial. This marked a paradigm shift from the previous convention of separation between adult and paediatric studies. As the pandemic unfolded, the investigators shaped the trial to address areas, such as the multisystem inflammatory syndrome (MIS-C)^1^ that is of particular relevance to children. Medicines with marketing authorisations for non-COVID indications in adults were repurposed and as the pandemic progressed, medicines were tested in children even though marketing authorisations were not in place. The investigators took care to consider issues around dosing and safety that were age-appropriate. The longstanding reluctance to include children at the same time as adults may in part have arisen from the perception of many investigators and regulators that paediatric studies are complex, time-consuming, or have added ethical issues. Having paediatricians engaged and involved in working groups and trial committees undoubtedly helped overcome such perceptions. The regulators, the UK Medicines and Healthcare Products Regulatory Agency (MHRA) in the case of RECOVERY, helped promote the need for concurrent studies in children alongside adults and worked closely with study teams, for example, to enable dose extrapolation where required. The RECOVERY study also benefited from having a research-experienced paediatric pharmacist closely involved from the conception of the study to advise on dosing, palatability and dispensing considerations.

The RECOVERY trial investigator’s efforts to include children from early in the pandemic enabled the first global trial of agents for MIS-C. If children had not been included in RECOVERY from the outset, it would have taken months to set up and start such a study. However, as it was, a large-scale study conducted across more than 170 hospitals in the UK was able to start recruiting children within a month of the start of the pandemic. The COVID-19 RECOVERY trial is example of what forward-thinking researchers can achieve with clinician engagement, regulatory support, political will and strong patient–public involvement. This experience sets a new standard, illustrating that wherever possible, and taking into account an informed consideration of the paediatric benefit–risk balance, adults and children can be included simultaneously in research and that the previous model of sequential investigation is not always necessary.

## Improving trial efficiency

The efficiency of randomised controlled trials has improved in recent years. These efficiencies encompass design innovations, improvements in operational aspects and public–patient involvement that ensure protocols are acceptable and feasible. A key lesson arising from the abrupt onset of the pandemic was the importance of building disaster preparedness into all three aspects.^2–6^

The RECOVERY protocol was designed to be implemented in routine clinical care. It used a risk-based approach approved by the UK regulatory agency, the MHRA, and national research ethics service (the Health Research Authority). This meant that governance was appropriate and clinicians were able to recruit after 20 min of online training without the need for full GCP certification or local delegation logs. The UK risk-based approach contrasts with the legal requirements put in place in some EU countries, such as the Netherlands, which required investigators to follow GCP guidelines strictly. While not suitable for early phase or higher risk drugs or trials, this experience suggests that many aspects of the conventional clinical trials process may have become over-regulated, over-burdensome and an obstacle to achieving rapid patient benefit, and as such, merit review.

Design innovations include the use of adaptive/platform trials, Bayesian approaches and co-enrolment to multiple trials. Investigators can use adaptive trial designs in early and late phase paediatric trials. These may involve master protocols with a platform (multiple therapies or combination of therapies) or a basket (different diseases) approach^7,8^ Investigators are also now able to use information technology to conduct “virtual” trials and simulations to inform trial design and operational aspects. Table 1 highlights important points to take into consideration for paediatric adaptive trials.

**Table 1:** Important points to consider in paediatric adaptive trial designs.

Item	Points to consider for paediatric trials
Early-phase clinical trials	•In children receiving chronic or long-term treatments protocols using an add-on investigational drug may improve consent rates, because the existing treatment will not be discontinued^8^ •Dose/exposure-response, including toxicity, can be different in children compared to adults^17,18,41–43^ •The choice of multiple vs single dose paediatric pharmacokinetic (PK) studies and use of dose escalation, including dose limiting toxicity, should be based on modelling and simulation, using innovative paediatric pharmacometrics, including physiology-based PK (PBPK) models^8,16,18,22,42^ •Consideration should be given to combining population PK extrapolated from adults or juvenile animals with PBPK models^8,16,22,42^ •Trial simulation can confirm optimal sparse sampling strategies, increase operational efficiency and improve trial success rates^7,8^ •Adaptive trial simulation may include scenarios for future pandemics or disasters which may impact trial recruitment, including recruitment bias caused by media reports and celebrity-endorsement or rejection of treatments^2,3,6,44^ •Paediatric research networks can help through centralised institutional review boards, electronic consent and standardised data capture^45^
Late-phase clinical trials	•Full, partial or no extrapolation from adult data may be used for paediatric trials, depending on the similarities and differences between children and adults in disease characteristics and predicted exposure responses^7,8,16–18,22,42,43^ •As with early-phase trials, pharmacometric modelling and simulation should be used with subsequent model validation^8,16,22,42^ •Late-phase paediatric trials should include PK/pharmacodynamic (PD) analyses to confirm exposure response^8,16,22,42,43^ •Continuous modelling and outcome adaptive randomisation using priors informed by adult studies and/or other indications (i.e. using Bayesian methods of prior knowledge and cumulative trial data) might be used^7,8^ •Evolving clinical trial arms and “promotion” of the control arm can be used as evidence builds (e.g. from external data such as concurrent adult studies or other paediatric age groups)^7,8^ •Pragmatic trials can take advantage of paediatric electronic health records and use centralised enrolment, randomisation, data collection and long-term follow-up reducing trial related workload for investigators^8^ •Paediatric research networks can help through centralised institutional review boards, electronic consent and standardised data capture^45^
Master protocols/platform trials	•May increase operational efficiency by using a harmonised paediatric research infrastructure^7,8^ •Can be designed to include new paediatric sub-studies and adapt to evolving treatment paradigms^7,8^ •May use a shared control group, thus reducing the overall sample size of children needed for a trial^7,8^ •Standardising paediatric efficacy/safety endpoints across studies and sites can be challenging^8,43,46^ •Early consultation with multiple, potential sponsors will increase the likelihood of agreement •May evaluate candidate compounds in gated phase I/II studies analysing paediatric PK and potential biomarkers for efficacy and safety before deciding on further paediatric drug development^8,43^ •Paediatric research networks can help through centralised institutional review boards, electronic consent and standardised data capture^45^

Another important key learning from COVID-19 trials has been the need for studies to adapt to emerging internal and external information.^4,5,9–14^ It is crucial to do so without undermining the integrity and validity of the study.^15^ For regulatory studies, this often means that the type-I-error is strictly controlled, and treatment effects are unbiased. The statistical framework has been extensively discussed but we wish to highlight that it is paramount that studies are designed with pre-planned opportunity for change in mind.^8^ Sufficient numbers of patients across the age spectrum are essential to ensure that one age group does not dominate the conclusions of the study, and to enable researchers to evaluate the consistency of the findings across age groups.

### Planning a paediatric study

Existing non-clinical and clinical data provide the evidence for protocol sections relating to the efficacy and safety of paediatric trials and the benefit–risk balance in children. Data synthesis and systematic reviews prior to and at the time of protocol writing help to address knowledge gaps. Research sponsors should consult clinical experts on practical aspects of the protocol. The protocol should include efficacy and safety endpoints which will trigger a withdrawal from the trial and where appropriate, the provision of rescue treatment.

We recommend that industry sponsors ensure that experts with experience of studies involving children and knowledge of country-specific aspects, as well as parent–patient organisations and young person’s advisory groups, review all paediatric protocols, informed consent or assent forms and operational aspects. Local expertise and knowledge are also required to take advantage of regional and national health care infrastructure for children who are not hospitalised. Table 2 lists examples of practical points to consider when writing a paediatric protocol.

**Table 2:** Examples of practical points to consider in writing a protocol for a multi-centre paediatric study.

Protocol item	Points to consider
PD endpoints	Ensure that pharmacodynamic (PD) endpoints for efficacy and/or safety have been validated for the paediatric study population and are clinically relevant^43^
Safety	Use the paediatric safety specification and paediatric risk management plan as the rationale for safety data collection and analyses (including Data Safety Monitoring Board reviews)^46^
Investigations (laboratory and vital signs)	Plan for differences in local paediatric laboratory reference values
	Ensure that age group-specific reference values for paediatric laboratory values and vital signs are used, and that results are interpreted by a paediatric specialist (e.g. paediatric electrocardiograms (ECG) should be read by a paediatric cardiologist)^47^
	Consider limitations on biosampling and interventional investigations in children^48^
	Ensure investigations and equipment used are adapted to the age group (e.g. an infant will usually not do a sitting blood pressure, the age appropriate practice is to take the blood pressure when the child is sleeping)
	Aim for integrating trial procedures and follow-up into routine paediatric care to keep disruption for children and their families to a minimum
Diagnostic criteria and standard treatments	Plan for differences in diagnostic criteria, treatments and comorbidities in children across different investigator sites

### Safety considerations

**Fig. 1:**
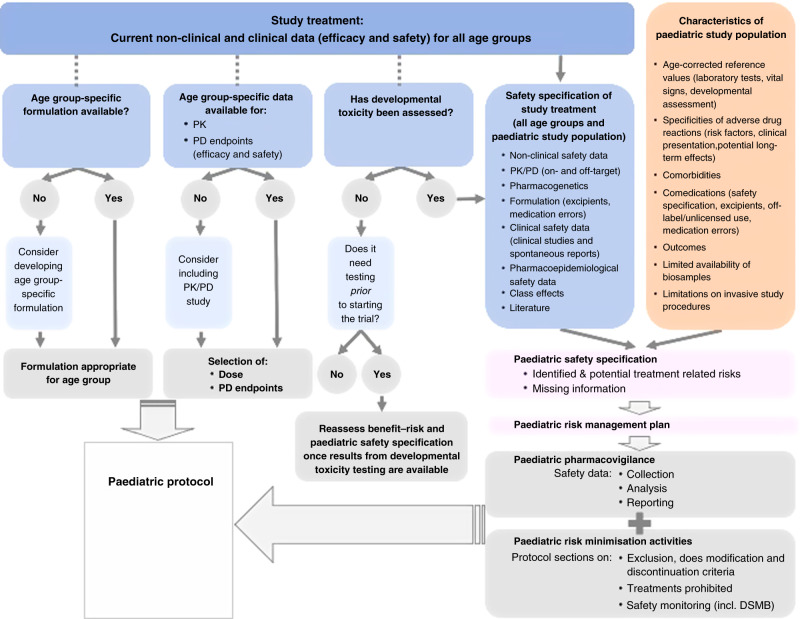
Issues to consider in the planning phase of a study including children.

Paediatric adverse drug reactions (ADR), differ both from those in adults, and also across age groups in children. Paediatric pharmacovigilance and risk minimisation are informed by the paediatric safety specification. The latter describes known paediatric ADR and potential treatment-related risks, and lists safety information that is missing, such as long-term effects on growth, cognitive development, or risk factors for known ADR.^3^ The paediatric safety specification is based on non-clinical and clinical data and the specificities of the paediatric target population (e.g. how ADR present in children, age-group-specific risk factors and confounders) and informs the paediatric risk management plan. Risk minimisation in paedatric protocols may, for example, relate to age-specific exclusion or dose modification criteria (Fig. 1).

### Innovative pharmacometrics

At the time of planning a paediatric trial, it is helpful to consider what age-specific pharmacokinetic (PK) and pharmacodynamic (PD) data are available. Where applicable modelling and simulation (e.g. physiologically based pharmacokinetic modelling) can be used to support dose selection.^8,16^ Investigators should consider the strengths and limitation of different PK models.^17–19^ Advanced PK methods (e.g. PBPK and population PK models), scavenged/opportunistic PK sampling techniques and innovative PK technologies (e.g. micro-dosing, salivary and urinary sampling, microneedle sampling, dried matrix spots, ultra-low and micro-volume assays) limit the frequency and volume of blood needed from children.^20,21^ Where relevant and feasible randomisation frameworks can be created to target systemic exposure instead of dose, thus adjusting for pharmacokinetic differences due to development.^17^ Where clinically relevant, pharmacogenetic testing might be used to take into consideration potential differences in gene expression between children and adults.^8^ Formulation development may need to be included at an early planning stage and formulations with no/few excipients should be favoured. Figure 1 illustrates some of the issues that we recommend considering in the planning phase of a paediatric study.

## Industry perspectives

The SARS-CoV-2 pandemic demonstrated the value of clear and timely communication between industry sponsors and investigative sites, ethics committees, and regulators to ensure the safety of children, the integrity of clinical data and the adoption of more efficient approaches. It brought to light operational issues that may mitigate pandemic-related issues, as well as bring about other improvements to future paediatric clinical trials. SARS-CoV-2 clearly illustrated the value of nimble paediatric trials that can rapidly yield meaningful results, as demonstrated by Gilead’s inclusion of participants aged 12 years and above from 15 March 2020 in Remdesivir studies (ClinicalTrials.gov Identifier: NCT04292730).

Over the past decade, industry sponsors have increasingly implemented novel approaches that reduce sample sizes, a cardinal consideration given that the population available for some paediatric trials may be small. Such novel approaches include extrapolation, platform trials, sequential, adaptive, *n* = 1 trials, Bayesian designs, and use of real-world data.^22–26^ These novel approaches, as well as moving away from placebo and active reference arms that are often underpowered, serve to limit the number of children having to visit clinical trial sites.

Another key learning has been the importance of swift and clear communication with investigative sites, research ethics committees, and regulatory bodies. Frequent updates regarding stopping and restarting of studies have been well received and reciprocated by sites with updates regarding their capacity to continue study participation. Flexibility, while maintaining trial integrity, is also critical. For example, incorporating fewer in-person assessments than initially planned into the statistical analysis plan available for review by regulatory bodies.

Before the pandemic, industry sponsors were increasingly considering decentralised clinical trial measures such as visits performed by telemedicine, roving or local healthcare practitioners, and data captured remotely. Such decentralised measures should improve the efficiency of future paediatric clinical trials after the pandemic resolves. Although the associated infrastructure and staffing requires investment, the many potential benefits to patients make this an important model moving forward.

Contingency planning, to mitigate the effects of a potentially prolonged duration and/or resurgence of the pandemic, is a key concept. Potential interventions include electronic consenting/assenting, home shipping and administration of the investigational product, remote source data verification and monitoring, local laboratory testing, increased flexibility in the timing of screening and endpoint windows for data that cannot be accurately obtained virtually, and processes to distinguish protocol deviations that are related to the pandemic from those that are not. Such mitigation planning will ideally ensure the continuity of current paediatric clinical trials, as well as future trials impacted by other natural disasters or political unrest. Moreover, sponsors can reduce the trial burden to children and their families by incorporating flexibility in timing and number of hospital visits. This is important as missing school or social activities is one of the main self-reported burdens to children when participating in trials.

In conclusion, many measures were previously recognised as ideal steps towards optimising and accelerating paediatric clinical trials, and thereby the approval of much-needed paediatric therapies. The pandemic provided opportunity to test many of these measures. Going forward, continued collaboration between industry sponsors, academia, regulatory bodies, patients and the public can build on the momentum created to advance the development of more efficient paediatric studies.

## Regulatory considerations

The pandemic instigated the adoption of new regulatory processes for COVID-19 research as well as other ongoing studies against a backdrop of significant social and medical impacts, redirection of hospital resources and quarantine conditions. Regulators also had to take into account the evolution of the disease, the development of and need to test new therapeutic interventions, emerging safety signals and changing contingencies associated with multiple waves of infection.^27^ Regulators were required to make decisions regarding temporary suspension, continuation or premature termination of pre-existing studies.

Regulators worked closely with healthcare partners and stakeholders to understand the effects on clinical trials and rapidly identify where flexibilities and additional support were necessary, without compromising participants’ safety or the scientific integrity of the studies. The European Medicines Agency (EMA), including its Good Clinical Practice Inspectors Working Group, the Clinical Trials Facilitation and Coordination Group and the Clinical Trials Expert Group, as well as the European Commission issued consensus guidance on the management of clinical trials during the COVID-19 pandemic. National regulatory agencies such as the MHRA in the UK and the US Food and Drug Administration issued similar guidance. The EMA also engaged with the World Health Organisation and national regulatory agencies around the world through the International Conference of Medicines Regulatory Authorities.

We searched ClinicalTrials.gov on 16 Oct 2020 to identify COVID-19 related studies from 1st December 2019 using the advanced search tools with the following keywords: COVID, COVID-19, SARS-CoV-2, severe acute respiratory syndrome, 2019-nCoV, 2019 novel coronavirus, Wuhan coronavirus. We would not class this as a systematic search but a review of key articles. We identified that to enable these studies, regulators and research ethics committees facilitated application submission processes (e.g. by creating dedicated mailboxes), and implemented procedures for rapid scientific advice, rolling reviews, and approvals (e.g. by creating ad hoc expert groups). Studies were also supported by the EMA rapid procedure through which a COVID treatment or vaccine Paediatric Investigation Plan can be agreed within a maximum of 20 days, compared to the usual 120 working day timeframe. In the UK, the MHRA established a dedicated COVID team to deal with queries and applications in an efficient, expedited manner. To facilitate global development, the EMA and FDA also produced a common commentary to guide the submission of Paediatric Investigation Plans in the EU and corresponding plans (IPSP) in the US.

COVID-19 vaccine studies will also need to recruit infants, children and young people. Vaccine studies represent a different level of risk to benefit for paediatric age groups as the recipients will be healthy at the time of receipt, and children are at very low risk for symptomatic COVID-19. However, although young children are not currently thought to be potent spreaders of COVID-19, they can still acquire the infection, and will become the young adults who are the major vectors in many countries.^28^

Three COVID-19 vaccines have now been approved for adults in some parts of Europe. However, though three PIPs have been approved, only one paediatric clinical trial has commenced and this limits inclusion to those aged 12–18 years (ClinicalTrials.gov Identifier: NCT04649151, searched 16 Jan 2021). All three vaccines received a PIP deferral, but the rationale is not publicly available and the consequences merit consideration. While paediatric vaccination initially appeared less critical as the disease burden is low in children, the increasing incidence of MIS-C and the urgent need for herd immunity, especially with the recent more contagious COVID mutants, suggests that paediatric vaccination may be an important and urgent public health consideration. The exclusion of children also raises the question of research equity. The delay or deferral of paediatric studies requires careful consideration at the outset as to whether this is truly justifiable on ethical or scientific grounds. We suggest that the inclusion of children at the outset should be the default approach unless there are clear and specific reasons that justify their exclusion, as has been suggested in the case of pregnant and breast-feeding women.^29^

A major challenge faced by research ethics committees regarding the approval of COVID-19 clinical trials related to the informed consent process in the context of an urgent and rapidly evolving global situation. The approaches adopted included witnessed consent, and physical separation of the clinical researcher seeking consent from patients in isolation. In the UK, the MHRA established a person-to-person interaction with the Health Research Authority to support rapid and efficient responses to research ethics queries. The MHRA also published blogs with risk information adapted trials and monitoring for paediatric trials.

For trials disrupted as a result of Covid-19, guidance was issued stating that regulatory and ethical requirements could be adapted, but should be properly justified, documented and approved by the corresponding regulatory authorities and research ethics committees. Other recommendations included performing a risk/benefit assessment, clear justification for selecting the participant population, Investigational Medicine Product mode of action, trial design and ethical implications. Investigators introduced a number of measures to mitigate risk to participants. These included consideration of the need for travel, performing laboratory tests at local centres and restricting follow-up visits and monitoring activities to those absolutely essential (e.g. for primary endpoint and safety reporting data). Other considerations were the maximum number of study participants that could attend at any one time at the research site, avoiding vulnerable participants meeting other patients and replacing site visits with video or phone calls. Regulators considered it acceptable to deliver an investigational medicinal product directly to the participants home, if necessary, accompanied by training for administration. Regulators also supported remote monitoring where appropriate and achievable without risking patient confidentiality.

In conclusion, international regulators demonstrated pragmatism and flexibility during the COVID-19 global pandemic while maintaining the safety of trial participants. Many actions indicate that regulators recognised the demands of an exceptional situation including the needs of children. These actions provide valuable insights to support safe but flexible and efficient regulatory innovations in the future.

## Infrastructure requirements

Public health systems recorded more than five million cases of COVID-19 worldwide in the first 10 months of the pandemic. At the time of writing, the duration of the pandemic remains uncertain. Infrastructural requirements specific to COVID-19 include virtual screening for symptoms, assessment of the risk-to benefit ratio for maintaining schedules for research visits, and maintaining a pool of research staff trained in preventive measures to optimise protocol adherence without risks to participant and staff safety.

The COVID-19 crisis has accelerated the application of measures that reduce person-to-person contacts, and support remote data gathering and study monitoring. These concepts are described in varying ways that include the terms decentralised clinical trials, direct‐to‐participant, and virtual studies. The technologies include wearables and personal sensors, and mobile or internet-based telemedicine and remote patient monitoring. Such approaches also reduce participant and staff burden. An example of a randomised clinical trial performed pre-COVID-19 with the use of a customised app is the PROPINE trial (EudraCT 2012-004326-16, sponsored by AIFA), which was conducted in Italy and involved children with nephrotic syndrome.^30^

Patient Generated Data, defined as “health-related information created, recorded, or gathered by or from patients, family members or other caregivers to help support and manage disease state” using new digital technologies and age-appropriate apps, are other approaches that can improve the efficiency of trials and the experience of research participation for children. Hybrid trials combining remote data collection and in-home with site visits can also lower participant patient burden but require paediatric trained staff to ensure age-appropriate care. The evaluation of patient compliance and protocol fidelity requires careful consideration.

Direct-to-patient shipping of trial investigational medicinal products requires defined standard processes. Also, sponsors should consider initiating more sites, validating local laboratories for routine care, keeping centralised laboratories for specialised tasks.

Ensuring the integrity, reliability and robustness of data generated in clinical trials is essential. There may also be a need to authorise patient enrolment through electronic informed consent (e-consent). ICH E6 (R2) requires that sponsors operating computerised trial data handling systems, validate these systems, and maintain an audit trail for initial entry of data and any subsequent changes, a security system to protect against unauthorised access and a list of the individuals authorised to create, access, modify or delete data.

In conclusion, COVID-19 studies have accelerated the introduction of new approaches. This provides opportunity for trial sponsors, research units and organisations, and regulatory bodies to incorporate budgetary and development of standard operating procedures into future planning.

### Emergency operational preparedness

Efficient operations are critical to recruitment, participant safety, protocol fidelity and quality data collection. Pandemic-related infection precautions may separate pharmacy and research staff from patients, increasing the research workload for the clinical team. For ongoing trials, sponsors may consider providing dedicated support.^31^ Sponsors considering protocol changes, as necessitated by the COVID-19 pandemic should engage with regulators and ethics committees as early as possible. Sponsors should be prepared to introduce changes to minimise immediate threats or limit exposure to the virus before filing an amendment and submitting this as soon as possible afterward.^2,3,32^ Table 3 summarises operational considerations for emergency preparedness using the example of the current COVID-19 global pandemic.

**Table 3:** Emergency preparedness: summary of key operational considerations for adaptive design trials using the example of the current COVID-A19 global pandemic.

Operational item	Points to consider
Harmonising similar protocols	Consider other COVID-19 studies for which the patient may be eligible for and harmonise the initial approach, and consenting process
Consent	Use electronic, video or verbal informed consent and assent by telephone^2^ Consider possibility of deferred consent and assent
Nursing and support staff	Train research nurses and bedside staff on more than one study
	Create resources for bedside staff on what (COVID-19) clinical trials, or observational studies their patients may be eligible for inclusion
	Integrate pharmacy members into the research team, and advise them early of potential intervention adaptations
	Create administration guides for each trial/intervention for the bedside staff
	Have research coordinators support bedside staff for reconciliation of investigational products
Study drug administration	Consider training parents or home visiting research nurses to administer the study drug
	If the study drug is to be administered at home, plan for supply chain and appropriate, safe storage and destruction (e.g. child-save containers, locked cabinet)
Visits	Conduct virtual visits; reduce hospital/site visits and select which patient reviews/tests can be done remotely, through home visits by research staff, or by a local health care provider/laboratory
Investigations, data collection	Utilise at-home testing (e.g. digital stethoscope, thermometer, otoscope, peak flow, blood glucose monitoring, dry blood spot for PK and other laboratory samples); use telephone follow-up, and mobile applications for data collection where possible to limit in-person study visits
Monitoring	Consider remote trial monitoring
Protocol amendments	Pre-emptively discuss trial adaptation plans with the research ethics board and other relevant committees; if possible, create a process for expedition of review

## Involving and engaging parents, carers and young adults

The involvement of children, young people and families in many aspects of research, including clinical trials in paediatric drug development, is now common. The inclusion of children and young people’s voices around the decision-making table helps to ensure that studies remain patient-centred and relevant.^33^ However, the COVID-19 pandemic altered the usual systems of study design and it is not clear if the involvement of children, young people and families kept pace. Establishing the type, and extent of activities involving children, young people and families for studies is difficult, as there are no specific areas on official registration sites such as ClinicalTrials.gov that mandates this information.^34^

To the best of our knowledge, no public–patient involvement has occurred, although we acknowledge this could have occurred without being explicitly mentioned. However, none of the studies involving children and young people that have already been completed that we identified on PUBMED (>1400 articles) and ClinicalTrials.gov (74 clinical trials) contained any mention that children, young people and families had been involved. Likewise, for ongoing studies, when ClinicalTrials.gov clinical study registry and the EU Clinical Trials Register for trials were searched. As previously mentioned, we would not class this as a systematic search but a review of key articles. We identified 113 clinical studies of COVID-19 treatment(s) involving patients less than 8 years of age, but no mention of the involvement of children, young people or families. However, we did identify studies examining the attitudes of parents about aspects of healthcare during the pandemic.^35^

The ongoing studies identified involve over 360,000 children and adults. Seven studies involved children from seven hours of age up to 17 years (*n* = 2410), with the remainder recruiting both children and adults. The interventions included drugs (*n* = 50), biological therapies (*n* = 20), devices (*n* = 9), diagnostic tests (*n* = 11), behavioural (*n* = 5), dietary (*n* = 5), and other studies (*n* = 13).

A large number of children participated in epidemiological studies. Many of these were developed rapidly, but some were pre-existing studies that were either re-activated or adapted to COVID-19. The World Health Organisation ISARIC (International Severe Acute Respiratory and Emerging Infections Consortium) is one of the leading epidemiological studies of COVID-19, recruiting 96,074 individuals from 562 sites across 42 countries (https://isaric4c.net/). Within the UK, this consortium recruited through the Clinical Characterisation Protocol for Severe Emerging Infections in the UK (CCP-UK) study. The protocol makes clear the tension when recruiting between an individual’s responsibilities to society, and the implications of this research for public health,^36^ but specific work with children, young people and families was not undertaken to determine what they thought about this (Professor M. G. Semple, CI CCP-UK, personal communication).

At the time of writing, there were 12 studies listed on clinicaltrials.gov related to COVID-19 and vaccinations involving children and young people.^37^ Of these, only three were studies administering vaccines to children. We contacted the three study teams and obtained a response from one, the team responsible for two studies in China. The researchers did not record child and family involvement in the development of these studies. We also sought input from a parent representative from the c4c network (DA) who is highly connected with patient involvement activities but was also not aware of any involvement of children and families in the design or the execution of any clinical trial on COVID-19 in children.

It is likely that in the early phases of the pandemic, the urgency of the trial design and deployment was likely the primary reason for the lack of children and family involvement. However, we wish to highlight that going forward, it is important to ensure that previous improvements in such involvement are not lost. We recommend promoting existing networks of children, young people and parent organisations who are knowledgeable about studies, and able to provide the rapid responses that industry and academia need in a rapidly changing situation such as that imposed by the COVID-19 pandemic. To provide access to these organisations, c4c is piloting a strategic feasibility advice service, which in addition to professional experts, also provides access to networks and individual parents and patients to provide advice on paediatric study design and conduct. We also recommend that relevant bodies such as the c4c network in Europe and the US Paediatric Trials Network develop clear guidance on involving children, young people and families in urgent and extreme circumstances, as well as “normal” conditions. Such guidance would need input from a range of children, young people, and families, as well as research-active paediatric clinicians familiar with the rapidly evolving science, and drug development pathway. Such guidance would also be helpful for funding bodies in setting standards for applicants, details for inclusion in trial and other registers, and identifying where additional research would be beneficial.

## Towards international harmonisation

Considerable progress has been made over the last decades in moving towards greater international harmonisation for paediatric research. In the European Union, the regulatory framework for paediatric medicines, the Paediatric Regulation, came into force in 2007.^38^ In 2017, the European Commission issued its 10-year report on the implementation of the Regulation. This showed that the number of medicines developed for children increased during this period but also revealed continuing challenges, especially concerning the development of medicines for diseases that only affect children and with features that differ in adults and children. The report also highlighted that the development and subsequent availability of paediatric medicines is slower than that for adult products. The Commission identified some areas for improvement in the current legal framework, and with the European Medicines Agency, developed a detailed plan to drive the development of medicines for children further in Europe. This takes into account suggestions made at a multi-stakeholder workshop convened by the European Commission and the European Medicines Agency on 20 March 2018 to discuss how to improve the implementation of the Paediatric Regulation.^39^ The actions are grouped according to the five thematic areas highlighted by the Commission in their ten-year report, one of which is to further strength international cooperation and increase interactions between the EMA Paediatric Committee and other stakeholders, including other regulators and paediatric clinical research networks, such as the European Network of Paediatric Research at the EMA (Enpr-EMA).^40^ We commend the conclusion that the involvement of Enpr-EMA with existing European paediatric networks and research organisations, such as Conect4children, European Reference Networks, European Paediatric Translational Research Infrastructure, European Network of Excellence for Paediatric Research (TEDDY) and other European reference networks, is an important area for prioritisation. We also urge identification and connection building across Europe to create centres of excellence in specialised areas. Finally, scientific mobility is an important component of capacity building; hence, we suggest that steps are taken to inform young researchers about the development of processes to improve international research harmonisation.

## Concluding remarks

COVID-19 has been a major disruptive force with significant impact on medical research. However, the crisis has also provided opportunity to introduce and test new ways of planning and delivering paediatric studies. We recommend that investigators, regulators, industry sponsors and clinicians take every opportunity to learn from these insights and experiences to improve future paediatric studies and accelerate improvements in the care of children.

## Supplementary information

Supplementary Appendix
