# Supplementary material for: Improving clinical paediatric research and learning from COVID-19: recommendations by the Conect4Children expert advice group
Source: Pediatr Res. 2021 Jun 7;91(5):1069–77. doi: 10.1038/s41390-021-01587-3 (PMC8184051; doi:10.1038/s41390-021-01587-3)
Supplement: Supplementary file 1 — Supplementary Appendix [file 41390_2021_1587_MOESM1_ESM.docx]

**Appendix**

| **last name author** | **prefix** | **first name author** | **affiliation** | **Country** |
| --- | --- | --- | --- | --- |
| Aurich |  | Beate | Robert Debré University Hospital, Paris | France |
| Bakhtadze |  | Sophia | Tbilisi State Medical University | Georgia |
| Bautista Sirvent |  | Francisco José | Hospital Niño Jesús, Madrid | Spain |
| Cabañas |  | Fernando | 1. Quironsalud Madrid University Hospital | Spain |
|  |  |  | 1. Biomedical Research Foundation, La Paz University Hospital-IDIPAZ, Madrid | Spain |
| Campbell |  | Lisa | Medicines and Healthcare Products Regulatory Agency MHRA | UK |
| Casanova |  | Michela | Fondazione IRCCS Istituto Nazionale dei Tumori di Milano | Italy |
| Charlton |  | Philippa | UCB Biopharma | Belgium |
| Crandall |  | Wallace | Eli Lilly and Company | USA |
| Eichler |  | Irmgard | European Medicine Agency EMA | The Netherlands |
| Fregonese |  | Laura | European Medicine Agency EMA | The Netherlands |
| Hawcutt |  | Daniel B. | 1. Department of Women’s and Children’s Health, University of Liverpool | UK |
|  |  |  | 1. NIHR Alder Hey Clinical research Facility, Alder Hey Children’s Hospital | UK |
| Iveli |  | Pablo | Bayer Aktiengesellschaft | Germany |
| Jaki |  | Thomas | Department of Mathematics and Statistics, Lancaster University and MRC Biostatistics Unit, University of Cambridge | UK |
| Jocic-Jakubi |  | Bosanka | 1. Clinical Center Nis, Pediatric Clinic, Child Neurology Dept. | Serbia |
|  |  |  | 1. Sultan Qaboos University Hospital, Dept. Pediatric Neurology, Muscat | Oman |
| Johnson |  | Mats | Gillberg Neuropsychiatry Centre, Sahlgrenska Academy, Gothenburg University | Sweden |
| Kaguelidou |  | Florentia | 1. Clinical Investigations Center, CIC1426, Robert Debré Hospital, Assistance Publique des Hôpitaux de Paris (APHP) | France |
|  |  |  | 2. Université de Paris | France |
| Karadag |  | Bülent | Marmara University Faculty of Medicine, Istanbul | Turkey |
| Kelly |  | Lauren E. | Department of Pediatrics and Child Health, University of Manitoba, Children's Hospital Research Institute of Manitoba | Canada |
| Lim |  | Ming | 1. Evelina London Children’s Hospital, Guys and St Thomas’ NHS Foundation trust | UK |
|  |  |  | 1. Department Women and Children’s Health, Faculty of Life Sciences and Medicine, King’s College London | UK |
| Modi |  | Neena | 1. Section of Neonatal Medicine, Department of Public Health and Primary Care, Imperial College London | UK |
|  |  |  | 1. Chelsea and Westminster NHS Foundation Trust, London | UK |
| Moreno |  | Carmen | Department of Child and Adolescent Psychiatry, Institute of Psychiatry and Mental Health, Hospital General Universitario Gregorio Marañón, School of Medicine, Universidad Complutense, IiSGM, CIBERSAM, Madrid | Spain |
| Neumann |  | Eva | Dr. Margarete Fischer-Bosch-Institute of Clinical Pharmacology, Stuttgart | Germany |
| Ollivier |  | Cecile | Aparito | The Netherlands |
| Oualha |  | Mehdi | 1. EA7323, University of Paris, Paris | France |
|  |  |  | 1. Pediatric Intensive Care Unit, AP-HP, Necker Hospital, Paris | France |
| Raffaeli |  | Genny | Department of Clinical Sciences and Community Health, University of Milan | Italy |
| Ramanan |  | Athimalaipet | University Hospitals Bristol NHS Foundation Trust | UK |
| Ribeiro |  | Maria Alexandra | NOVA Medical School, Faculdade de Ciências Médicas, Universidade Nova de Lisboa | Portugal |
| Roilides |  | Emmanuel | 1. Aristotle University of Thessaloniki | Greece |
|  |  |  | 2. Hippokration Hospital | Greece |
| Rojas Pablo | de | Teresa | Accellerate | Spain |
| Rubio San Simón |  | Alba | Hospital Niño Jesús, Madrid | Spain |
| Ruperto |  | Nicolino | IRCCS Istituto Giannina Gaslini | Italy |
| Scarpa |  | Maurizio | Regional Coordinating Center for Rare Diseases, University Hospital, Udine | Italy |
| Schwab |  | Matthias | 1. Dr. Margarete Fischer-Bosch - Institut für Klinische Pharmakologie | Germany |
|  |  |  | 1. Department of Clinical Pharmacology, University Hospital, Tübingen | Germany |
| Siapkara |  | Angeliki | Medicines and Healthcare Products Regulatory Agency MHRA | UK |
| Singh |  | Yogen | 1. Cambridge University Hospitals NHS Foundation Trust | UK |
|  |  |  | 1. University of Cambridge School of Clinical Medicine | UK |
| Smits |  | Anne | 1. Neonatal intensive care unit,University Hospitals Leuven, Leuven | Belgium |
|  |  |  | 1. Department of Development and Regeneration, KU Leuven, Leuven | Belgium |
| Striano |  | Pasquale | 1. IRCCS Istituto Giannina Gaslini | Italy |
|  |  |  | 2. University of Genova, Genova | Italy |
| Urru |  | Silvana A.M. | S. Chiara Hospital Trento | Italy |
| Vivarelli |  | Marina | Division of Nephrology and Dialysis, Department of Pediatric Subspecialties, Bambino Gesù Pediatric Hospital IRCCS, Rome | Italy |
| Wildt | de | Saskia | 1. Department of Pharmacology and Toxicology, Radboud Institute for Health Sciences, Radboudumc, Nijmegen | The Netherlands |
|  |  |  | 1. Intensive Care and Department of Paediatric Surgery, Erasmus MC Sophia Children’s Hospital, Rotterdam, the Netherlands | The Netherlands |
| Zivkovic |  | Zorica | 1. Children's Hospital for Lung Diseases & Tb Clinical Center Dr Dragiša Mišovic, Belgrade | Serbia |
|  |  |  | 1. Faculty of Pharmacy Novi Sad, Business Academy, Novi Sad | Serbia |
